# Susceptibility of HPV-18 Cancer Cells to HIV Protease Inhibitors

**DOI:** 10.3390/v16101622

**Published:** 2024-10-17

**Authors:** Lilian Makgoo, Salerwe Mosebi, Zukile Mbita

**Affiliations:** 1 Department of Biochemistry, Microbiology and Biotechnology, University of Limpopo, Private Bag X 1106, Sovenga, Polokwane 0727, South Africa; lilian.makgoo@ul.ac.za; 2 Department of Life and Consumer Sciences, University of South Africa, Private Bag X06, Florida 1710, South Africa; mosebs@unisa.ac.za

**Keywords:** cervical cancer, human papillomavirus, HIV-PIs, cytotoxicity, apoptosis, cell cycle

## Abstract

Cervical cancer cases continue to rise despite all the advanced screening and preventative measures put in place, which include human papillomavirus (HPV) vaccination. These soaring numbers can be attributed to the lack of effective anticancer drugs against cervical cancer; thus, repurposing the human immunodeficiency virus protease inhibitors is an attractive innovation. Therefore, this work was aimed at evaluating the potential anticancer activities of HIV-PIs against cervical cancer cells. The MTT viability assay was used to evaluate the effect of HIV protease inhibitors on the viability of cervical cancer cells (HeLa) and non-cancerous cells (HEK-293). Further confirmation of the MTT assay was performed by confirming the IC_50_s of these HIV protease inhibitors on cervical cancer cells and non-cancerous cells using the Muse™ Count and Viability assay. To confirm the mode of death induced by HIV protease inhibitors in the HPV-associated cervical cancer cell line, apoptosis was performed using Annexin V assay. In addition, the Muse™ Cell Cycle assay was used to check whether the HIV protease inhibitors promote or halt cell cycle progression in cervical cancer cells. HIV protease inhibitors did not affect the viability of non-cancerous cells (HEK-293), but they decreased the viability of HeLa cervical cancer cells in a dose-dependent manner. HIV protease inhibitors induced apoptosis in HPV-related cervical cancer cells. Furthermore, they also induced cell cycle arrest, thus halting cell cycle progression. Therefore, the use of HIV drugs, particularly HIV-1 protease inhibitors, as potential cancer therapeutics represents a promising strategy. This is supported by our study demonstrating their anticancer properties, notably in HPV-associated cervical cancer cell line.

## 1. Introduction

Cervical cancer (CC) continues to pose a global public health challenge despite the existence of preventative measures. This type of cancer is characterized by the abnormal proliferation of glandular or squamous cells that line the cervix, primarily due to infection with high-risk HPV strains [1]. In South Africa, cervical cancer is recorded as the second most frequently diagnosed cancer in women [2]. In 2020, cervical cancer accounted for 342,000 fatalities, making it the fourth most prevalent cancer among women worldwide [3]. These alarming statistics highlight the urgency of intensifying efforts to prevent and manage CC, especially in low- and middle-income countries. Additionally, the current treatments available for advanced CC often yield unsatisfactory outcomes and have the potential to induce significant side effects, highlighting the critical need for the development of more efficient therapeutic interventions [4,5,6].

Drug discovery plays a crucial role in the development of new pharmaceutical products, as it involves the transformation of a mere concept into a market-ready drug molecule. However, the drug discovery process is not only time-consuming but also expensive, with an average timeline of 12 to 15 years for a drug to be officially approved [7,8]. To overcome issues with drug discovery, drug repurposing (DR) is increasingly being recognized as a promising alternative [9]. The primary benefit of employing the drug repurposing strategy lies in the pre-existing knowledge available regarding the known drug’s safety profile, clinical application, and manufacturing processes [10]. A classical example of DR is zoledronic acid, an anti-resorptive drug used to treat osteoporosis, in oncology, zoledronic acid is used for bone metastases [11,12]. Furthermore, nelfinivir, an antiretroviral medication specifically developed to combat the Human Immunodeficiency Virus (HIV), is currently being evaluated in phase I clinical trials as a potential therapeutic option for the management of solid tumors [13]. In light of these results, it is clear that repurposing HIV protease inhibitors (HIV-PI) to treat cervical cancer could be a promising strategy.

A class of antiviral agents known as HIV-PIs, incorporated into highly active antiretroviral therapy (HAART), has exhibited both antitumor and antiangiogenic effects in experimental settings devoid of HIV-1 or immune cells [14,15]. Since then, various studies have indicated that protease inhibitors (PIs) could exhibit antitumor effects, though these effects differ in magnitude. Specifically, ritonavir, saquinavir, and nelfinavir have been shown to cause growth arrest and apoptosis in multiple myeloma cells when tested in vitro [16]. Moreover, evidence suggests that indinavir reduces the invasive capacity of hepatocarcinoma cell lines in vitro and contributes to the delayed growth of these tumors in a nude mouse model in vivo [17]. In the context of HIV protease inhibitors that may demonstrate activity against cervical disease linked to HPV, lopinavir has emerged as the most effective compound when tested on SiHa and CaSki cervical cancer cell lines [18]. Findings from pre-clinical studies propose that applying lopinavir directly to the cervix through a vaginal pessary or gel formulation may effectively target HPV-associated cervical dysplasia [19]. Despite great progress, the use of lopinavir in HIV patients still faces many challenges, such as inducing insulin resistance and lipodystrophy [20]. Compared to lopinavir/ritonavir, atazanavir/ritonavir offers better viral suppression with fewer side effects [20,21,22]. Nonetheless, the extent to which atazanavir, either alone or in combination with ritonavir, can replicate the apoptotic and cell cycle arrest effects of lopinavir in HPV 18-associated cervical cancer cells remains insufficiently understood. Therefore, the current study focuses on assessing the anticancer properties of atazanavir, utilized independently and in combination with ritonavir (Ritoataz) in HPV-18 cervical cancer cells (HeLa). This assessment is conducted in relation to lopinavir, whose mechanisms of action in cervical cancer are already established.

## 2. Materials and Methods

Human cervical cancer cells (HeLa [CCL-2™] and non-cancerous cells (HEK-293 [CRL-1573]) were donated by the Universities of Johannesburg (UJ) and Pretoria (UP). Fetal bovine serum (FBS) and Dulbecco’s modified eagle medium (DMEM) were procured from Hyclone (South Logan, UT, USA). An antibiotic mixture of penicillin and streptomycin (Pen-Strep), MTT [3-(4,5-dimethylthiazol-2-yl)-2, 5-diphenyltetrazolium bromide] and phosphate-buffered saline (PBS) were all purchased from ThermoFisher Scientific (Waltham, MA, USA) while dimethyl sulfoxide (DMSO) was obtained from Sigma-Aldrich (St. Louis, MA, USA). The Muse^®^ (Merck-Millipore, Darmstadt, Germany) assay kits (Muse^®^ Cell Cycle Assay, Muse^®^ Count and Viability Assay, Muse^®^ Annexin V and Dead Cell Assay) and lopinavir were all purchased from Merck-Millipore (Darmstadt, Germany). Aluvia and Ritoataz tablets were all purchased from a local pharmacy. UHPLC-MS/MS was used to isolate atazanavir from Ritoataz tablets which contain 300 mg of atazanavir and 100 mg of ritonavir.

Ritoataz tablets and Aluvia tablets (50 mg ritonavir and lopinavir 200 mg) were crushed and dissolved in 80% ethanol to make a stock solution of 10 mg/mL under the assumption that each tablet consisted solely of two HIV-PIs. The stock solution in 10 mg/mL was then converted to micromolar using the equation below:

(Micromolar) μM = [1000/Molecular weight (in g/mol)] × provided μg/mL

The concentration of the combined HIV protease inhibitors in 1:3 ratio found in Ritoataz tablets and 1:4 ratio found in Aluvia tablets were used in comparison to the pure HIV protease inhibitors. All the experiments were performed in triplicates of three independent experiments; the graphical data were analyzed using GraphPad Prism Version 9.0 Statistical Software using the Tukey Kramer Multiple Comparison Test. The differences between the two sets of data were considered significant at *p* ≤ 0.05, comparing each sample with the untreated control.

### 2.1. Cell Culture

Following the manufacturer’s instructions, cell lines were cultured in DMEM supplemented with 10% FBS, and culture flasks were then incubated at 37 °C in a humidified incubator containing 5% CO_2_.

### 2.2. In Vitro Cytotoxicity Assays

For in vitro cytotoxicity assay, two assays were utilized, which are the MTT assay and Muse^®^ Count and Viability assay. MTT was used to determine the cell viability of cervical cancer cells in response to treatment with pure HIV protease inhibitors (lopinavir and atazanavir), commercially available HIV protease inhibitor tablets (Aluvia and Ritoataz), and curcumin, which served as a positive control. MTT assay was then followed by the Muse^®^ Count and Viability assay to confirm the calculated IC_50_ values.

#### 2.2.1. MTT Assay

The effects of pure HIV protease inhibitors (lopinavir and atazanavir), commercially available HIV protease inhibitor tablets (Aluvia and Ritoataz) on the viability of cervical cancer cell line (HeLa) as well as the non-cancerous HEK-293 were determined using the MTT assay. In brief, cells were cultured in flasks until 80% confluency and counted using Countess Cell Counter (ThermoFisher Scientific, Waltham, MA, USA). Cells were seeded in 96-well plates at 1 × 10^3^ cells/well and incubated overnight to allow the cells to attach. Cells were then exposed to various concentrations of lopinavir and atazanavir (0, 30, 60, 90, 120, 150 and 180 µM) and solvent control [lopinavir: 0.5% DMSO and atazanavir: ethanol (EtOH) 0.6%] for a duration of 24 and 48 h with atazanavir and 24–72 h with lopinavir in order to search for the concentration that can inhibit 50% cell viability (IC_50_). Ritoataz tablets (atazanavir 300 mg and 100 mg ritonavir) and Aluvia tablets (lopinavir 200 mg and 50 mg ritonavir) were crushed into powder and dissolved in 80% ethanol to make a stock solution of 10 mg/mL. Subsequent to stock preparation, cells were exposed to various concentrations of Aluvia and Ritoataz pills (0, 30, 60, 90, 120, 150, and 180 µM) and EtOH (1.9% in Aluvia and 2% in Ritoataz pills) for 24 and 48 h. Lastly, the cells were exposed to different concentrations of the positive control, curcumin (0, 10, 20, 30, 40, 50, 60 µM) for 24 h. Following incubation, an MTT assay was performed according to Laka et al. [23].

#### 2.2.2. Muse^®^ Count and Viability Assay

Further confirmation of the IC_50_ values calculated from the MTT assay was achieved by conducting an analysis using the Muse™ Count and Viability Kit. The HeLa cells, as well as the non-cancerous HEK-293 cells, were seeded at 1 × 10^3^ cells/well in 24-well plates overnight. Following incubation, a sterile 1 × PBS wash was performed on the cells, followed by treating the cells for 24 h with the IC_50_ of curcumin (40 µM), 72 h with 0.5% DMSO and the IC_50_ of lopinavir (90 µM), 48 h with 0.6% EtOH and the IC_50_ of atazanavir (150 µM), 48 h with 1.9% EtOH and the IC_50_ of Aluvia pills (150 µM); and lastly, 2% EtOH and the IC_50_ of Ritoataz pills (150 µM). Following treatment exposure, 1 × 10^6^ cells/mL were kept for analysis, Muse^®^ Count and Viability assay was performed according to Makgoo et al. [24].

### 2.3. Cell Cycle Analysis

To assess the effect of lopinavir (90 µM), atazanavir (150 µM), Aluvia pills (150 µM), Ritoataz pills (150 µM) and curcumin (positive control) on the cell cycle phases distribution, the Muse™ Cell Cycle Kit was used. Briefly, the HeLa cells were cultured in 12-well plates at 1 × 10^5^ cells/well and allowed to attach overnight. The cells were then synchronized for 12 h and then treated with the IC_50_s of the HIV protease inhibitors and the positive control. Following exposure to different treatments, cells were collected and washed with sterile 1 × PBS, followed by fixation in 70% ethanol for at least 3 h at −20 °C. Subsequent fixation, 1 × 10^7^ cells/mL were kept for analysis; cell cycle protocol was performed according to Laka et al. [23] and analyzed using the Muse^®^ Cell Cycle Analyzer.

### 2.4. Muse^®^ Annexin V and Dead Cell Assay

To determine the mode of death induced by HIV protease inhibitors (lopinavir and atazanavir), commercially available HIV protease inhibitor tablets (Aluvia and Ritoataz) and curcumin in HeLa cells, the Muse™ Annexin V and Dead Cell Kit were used following the manufacturer’s instructions. Briefly, HeLa cells were cultured in 24-well plates at 1 × 10^4^ cells/well overnight and treated with the IC_50_s of the HIV protease inhibitors and the positive control. Following treatment exposure, 1 × 10^5^ cells/mL were collected by centrifugation at 300× *g* for 10 min and used for analysis. The percentage of apoptotic cells was quantified and analyzed using the Muse^®^ Cell Analyzer.

## 3. Results

### 3.1. Cytotoxic Effects of Pure HIV Protease Inhibitors and HIV Protease Inhibitor Tablets against HeLa Cervical Cancer Cells

The cytotoxic effects of pure HIV protease inhibitors, namely, lopinavir and atazanavir, as well as the HIV protease inhibitor tablets, Aluvia and Ritoataz, were assessed against the HeLa human cervical cancer cells. This was accomplished through the use of the MTT assay and the Muse Count and Viability assay. The aforementioned tests are extensively employed in in vitro toxicity investigations to identify cytotoxic and other adverse impacts on cellular viability after exposure to experimental substances [23,24]. Therefore, to prevent the overestimation or underestimation of the toxicity of pure HIV protease inhibitors and HIV protease inhibitor tablets on the viability of human cervical cancer cells, both of these assays were employed to determine cell viability.

**Figure 1:**
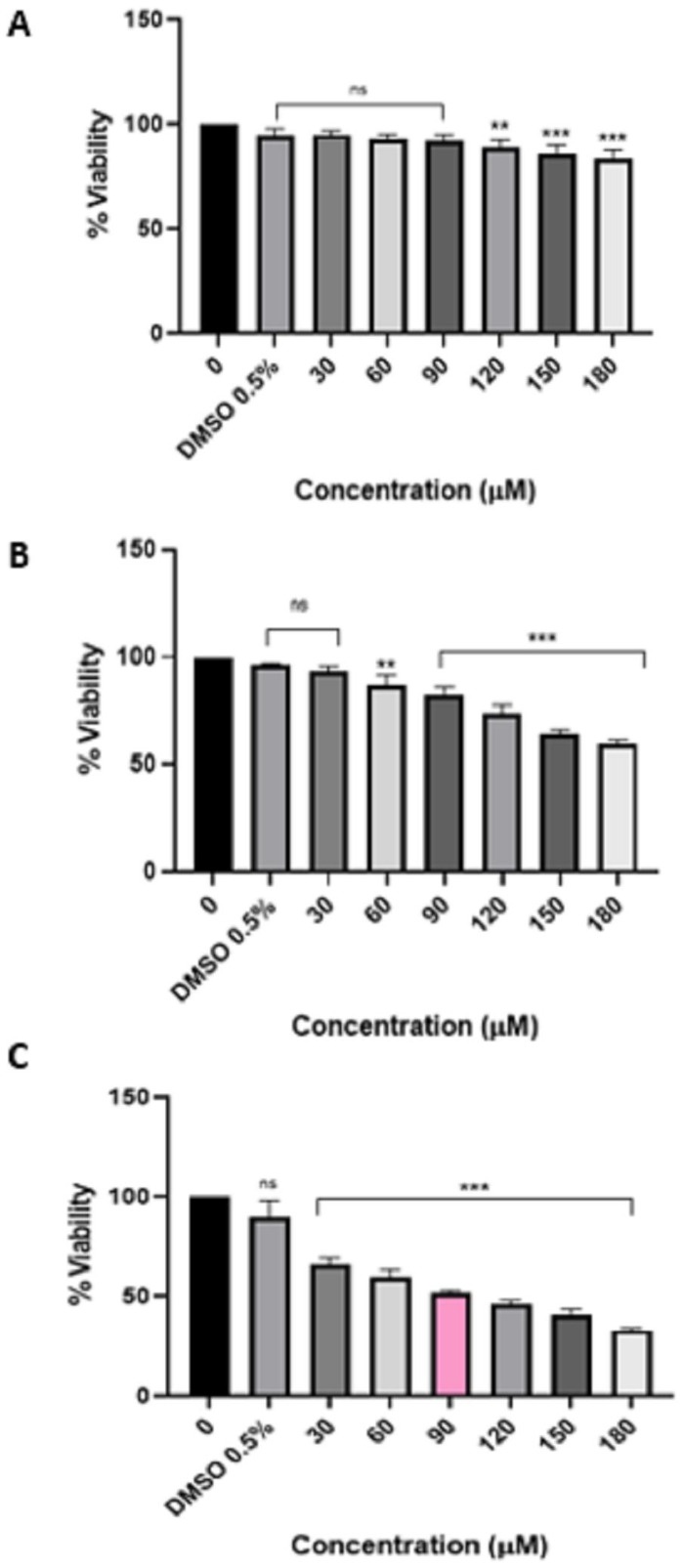
Effect of lopinavir HIV protease inhibitor on the viability of HeLa cells after 24 h treatment (**A**), 48 h treatment (**B**), and 72 h treatment (**C**). Lopinavir significantly (*p* ≤ 0.01 **/*p* ≤ 0.001 ***) reduced the viability of HeLa cells in a time and concentration-dependent manner. “ns” indicates a non-significant effect.

**Figure 2:**
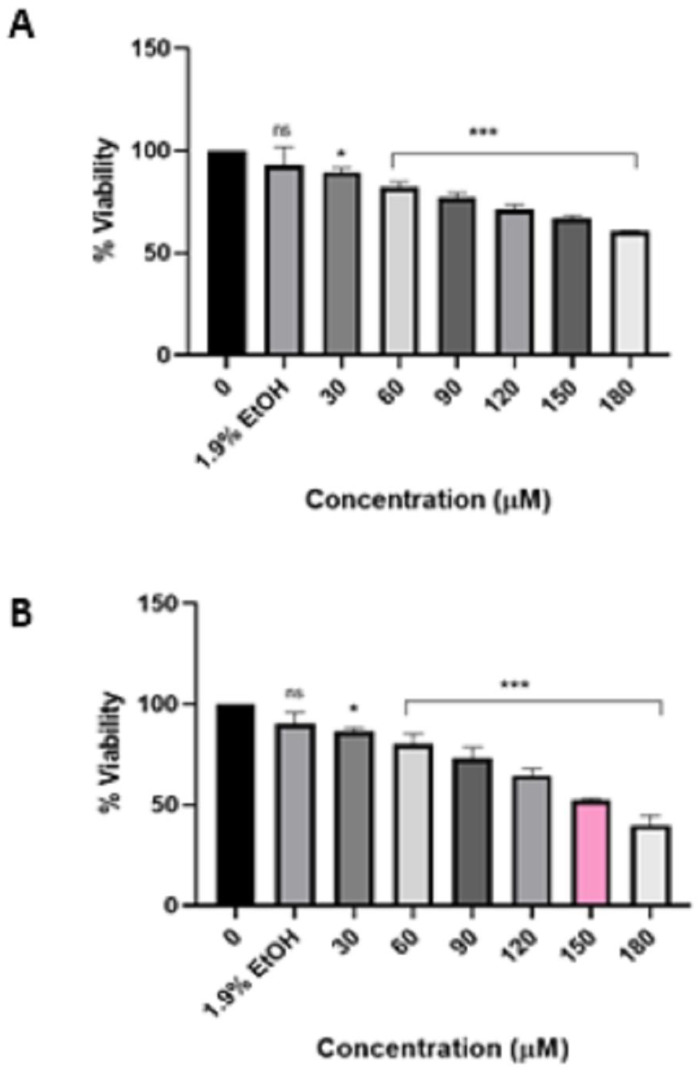
Effect of Aluvia HIV protease inhibitor tablets on the viability of HeLa cells after 24 h treatment (**A**) and 48 h treatment (**B**). Aluvia tablets significantly (*p* ≤ 0.05 */*p* ≤ 0.001 ***) reduced the viability of HeLa cells in a time and concentration-dependent manner. “ns” indicates a non-significant effect.

According to the findings on the viability of HeLa cells after treatment with pure HIV protease inhibitor lopinavir (Figure 1) and Aluvia tablets (Figure 2), it can be inferred that HeLa cells exhibit greater sensitivity to Aluvia tablets as compared to pure lopinavir. This conclusion is based on the calculated IC_50_ values, which indicate that after a 72 h treatment with lopinavir, the IC_50_ value for HeLa cells was determined to be 90 µM. In contrast, after a 48 h treatment with Aluvia tablets, the IC_50_ value against the HeLa cells was calculated to be 150 µM.

**Figure 3:**
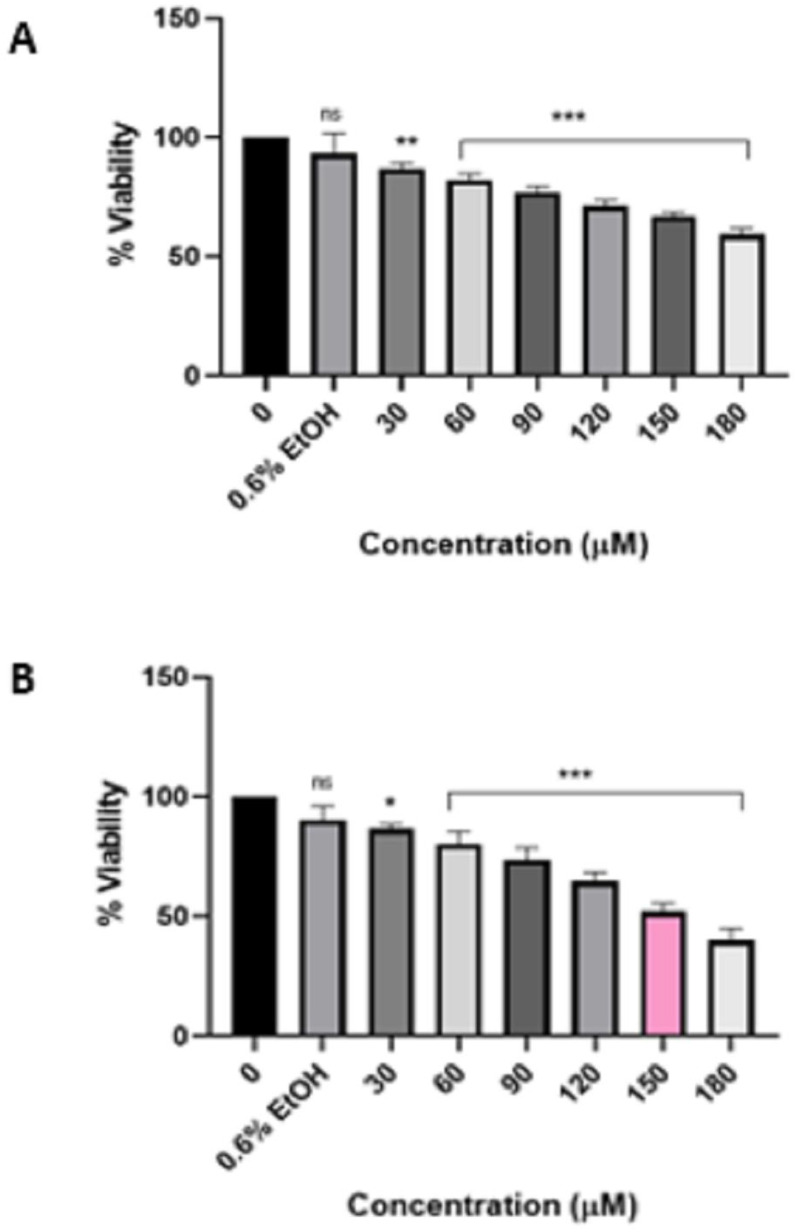
Effect of atazanavir HIV protease inhibitor on the viability of HeLa cells after 24 h treatment (**A**) and 48 h treatment (**B**). Atazanavir significantly (*p* ≤ 0.05 */*p* ≤ 0.01 **/*p* ≤ 0.001 ***) reduced the viability of HeLa cells in a time and concentration-dependent manner. “ns” indicates a non-significant effect.

**Figure 4:**
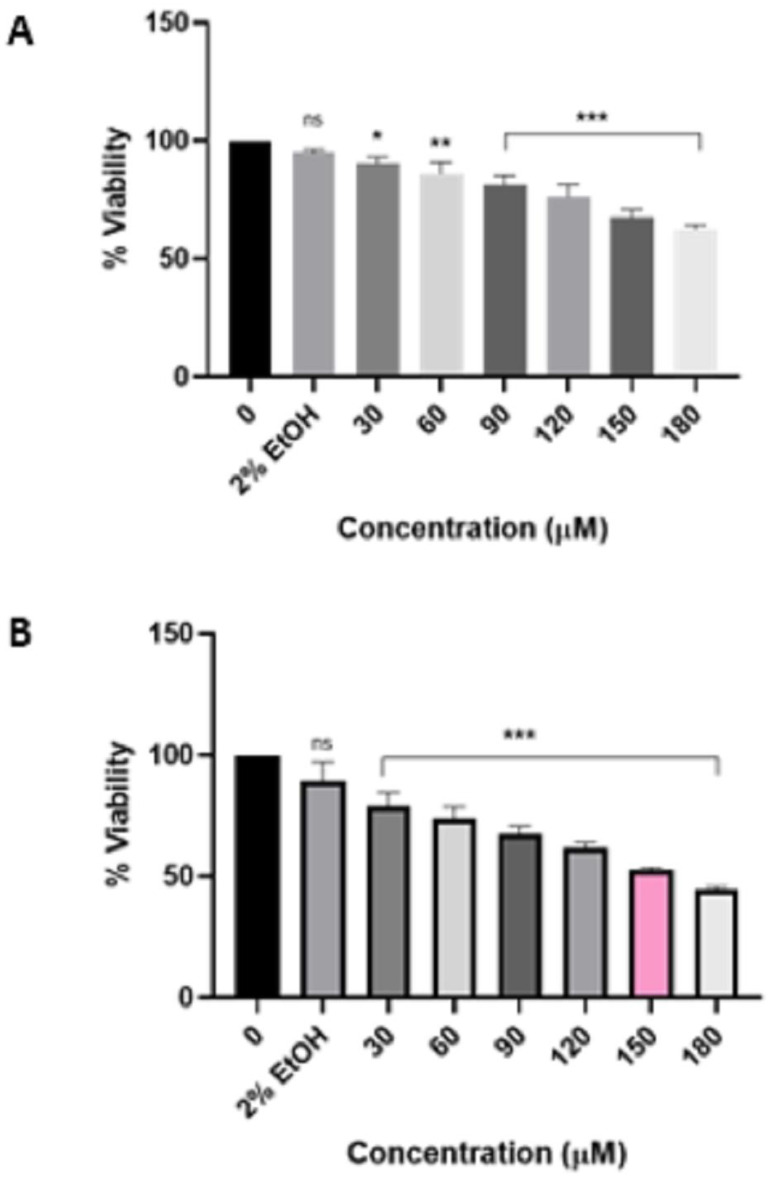
Effect of Ritoataz HIV protease inhibitor tablets on the viability of HeLa cells after 24 h treatment (**A**) and 48 h treatment (**B**). Ritoataz tablets significantly (*p* ≤ 0.05 */*p* ≤ 0.01 **/*p* ≤ 0.001 ***) reduced the viability of HeLa cells in a time and concentration-dependent manner. “ns” indicates a non-significant effect.

**Figure 5:**
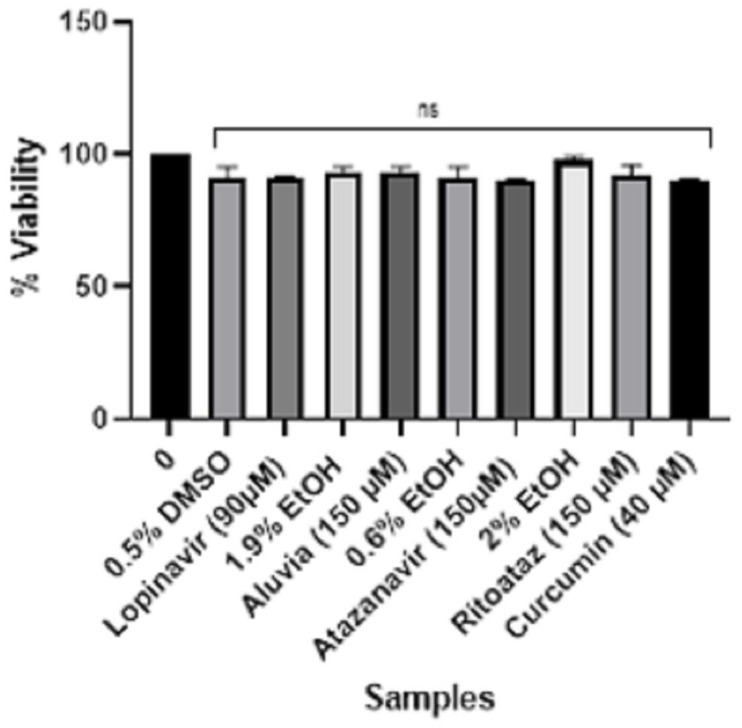
An analysis of the cytotoxicity of pure HIV protease inhibitors and HIV protease inhibitor tablets against HEK-293 human embryonic kidney cells. Statistically, there was no significant difference (ns) between the treated and untreated control groups.

**Figure 6:**
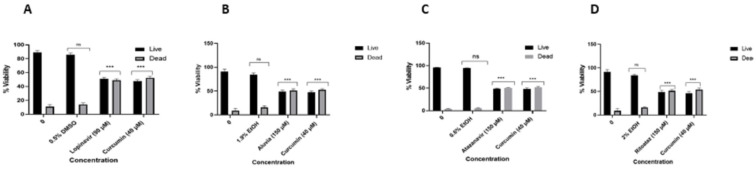
Confirmation of percentage viability of HeLa cells in response to treatment with lopinavir (**A**), Aluvia tablets (**B**), atazanavir (**C**), and Ritoataz tablets (**D**). All the HIV drugs significantly (*p* ≤ 0.001 ***) inhibited the viability of HeLa cells in vitro when compared to the untreated control. “ns” indicates a non-significant effect.

**Figure 7:**
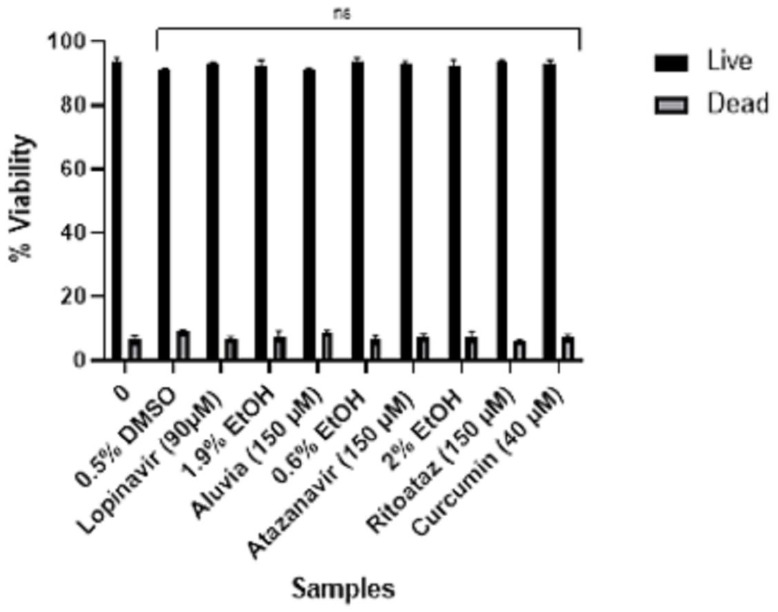
Confirmation of percentage viability of HEK-293 cells in response to treatment with pure HIV protease inhibitors and HIV protease inhibitor tablets. Compared to the untreated control, the difference was found to be statistically non-significant (ns).

**Figure A1:**
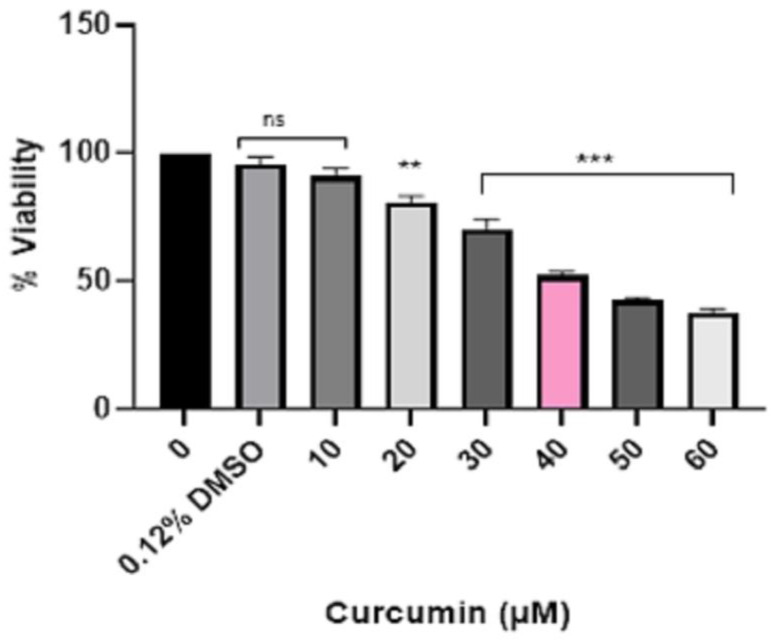
Effect of curcumin on the viability of HeLa cells after 24 h treatment. Curcumin significantly (*p* ≤ 0.01 **/*p* ≤ 0.001 ***) reduced the viability of HeLa cells in a time and concentration-dependent manner. “ns” indicates a non-significant effect.

Furthermore, upon scrutinizing the effect of the pure HIV protease inhibitor atazanavir (Figure 3) and Ritoataz tablets (Figure 4), it is evident that the HeLa cells exhibit identical responses to both drugs, as the IC_50_ value was ascertained to be 150 µM following a 48 h treatment. As a positive control, curcumin (Figure A1) had an IC_50_ value of 40 µM after 24 h treatment of HeLa cells. In addition, it is worth noting that based on the MTT assay results, all these drugs did not have a significant effect on the viability of non-cancerous cells (Figure 5). Moreover, the Muse Count and Viability assay corroborated the findings of the MTT assay and validated the accuracy of the IC_50_ values. The results of the Muse Count and Viability analysis are presented in Figure 6. The Muse Count and Viability assay provided further evidence that the tested drugs do not exhibit cytotoxic effects on non-cancerous HEK-293 cells, as illustrated in Figure 7.

When a cell is subjected to stress or has incurred DNA damage, it undergoes cell cycle arrest, a condition frequently triggered by the treatment of cancer cells with anticancer agents [25,26]. In cases of severe damage, mitochondrial mechanisms are triggered to convert the cell cycle arrest signal into an apoptosis signal through p53 [27]. It is evident that for cell cycle arrest and apoptosis to occur, the drug in question (HIV-PIs) must exhibit cytotoxic effects on the cells under investigation. Our findings revealed that HIV-PIs under investigation lacked significant cytotoxic effects on HEK-293 cells. Therefore, we did not see the necessity to test these compounds for their ability to cause cell cycle arrest and apoptosis in HEK-293 cells. Furthermore, since the HIV protease inhibitors under examination demonstrated cytotoxicity toward HeLa cells but not toward HEK-293 cells, we directed our efforts toward HeLa cells to elucidate the mechanisms used by these protease inhibitors to inhibit cell viability.

### 3.2. Pure HIV Protease Inhibitors and HIV Protease Inhibitor Tablets Induce Apoptosis in HeLa Cervical Cancer Cells

**Figure 8:**
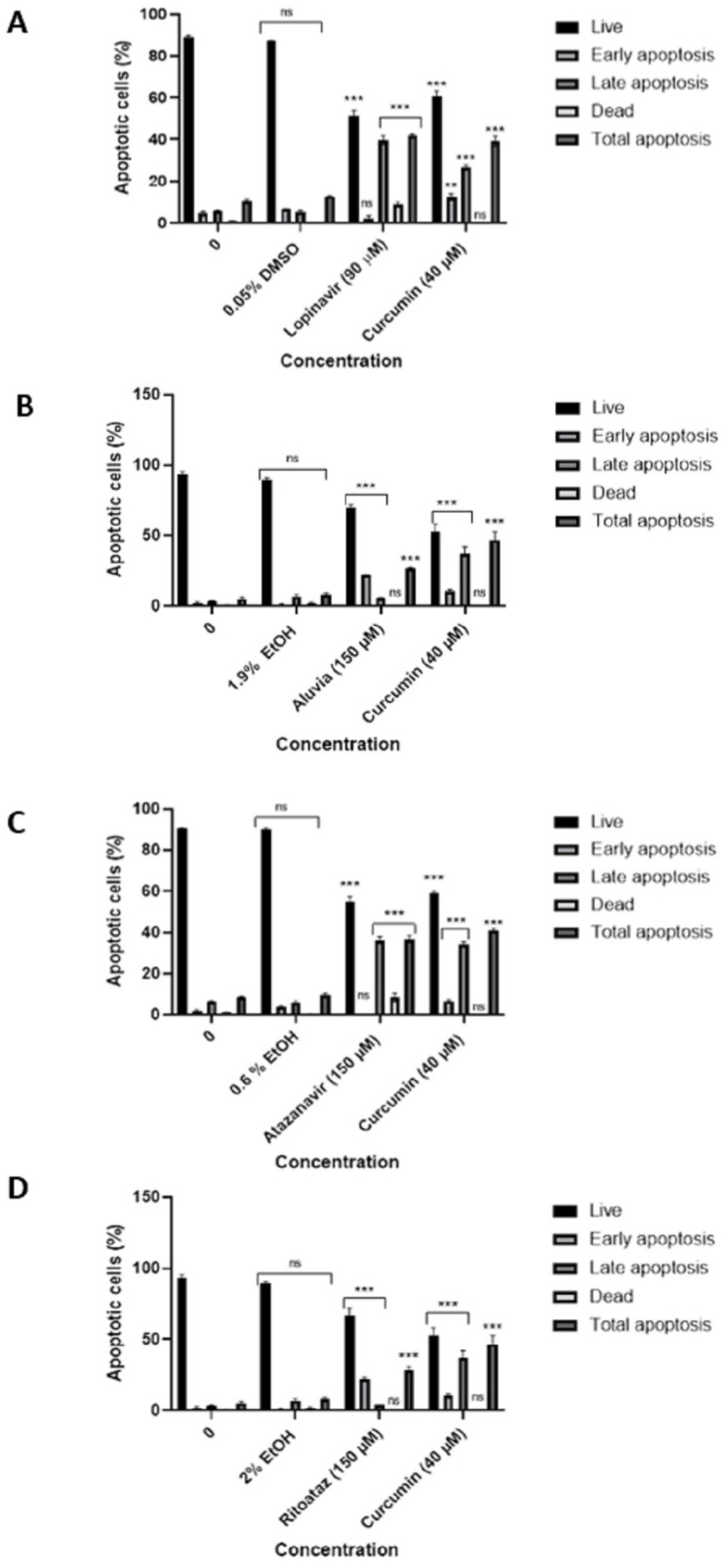
Average % apoptosis in lopinavir (**A**), Aluvia (**B**), atazanavir (**C**), and Ritoataz (**D**)-treated HeLa cells. HIV protease inhibitors and curcumin (40 μM) significantly (*p* ≤ 0.01 **/*p* ≤ 0.001 ***) induced apoptosis cell death in HeLa cells relative to the untreated control. “ns” indicates a non-significant effect.

In order to determine the specific type of cell death that is induced by pure HIV protease inhibitors and HIV protease inhibitor tablets in HeLa cells, the Muse™ Annexin V and Dead assay was employed. The results obtained from the Muse™ Annexin V and Dead Cell analysis indicated that the treatment of HeLa cells with 90 µM of lopinavir (Figure 8A), 150 µM of Aluvia tablets (Figure 8B), 150 µM of atazanavir (Figure 8C), and 150 µM of Ritoataz tablets (Figure 8D) resulted in a significant (* *p* ≤ 0.05) induction of apoptosis in HeLa cells. Upon comparison of the apoptotic effects induced by lopinavir and its counterpart tablets, Aluvia, it was observed that lopinavir elicited a greater degree of apoptosis (Table 1). Moreover, upon comparing the overall apoptosis triggered by atazanavir and its corresponding tablets, it was observed that Ritoataz tablets induced less apoptosis than atazanavir (Table 1). The findings suggest that the standalone HIV protease inhibitors trigger a higher rate of apoptosis compared to the tablets that contain ritonavir. It has been shown previously that treatment of HeLa cells with ritonavir inhibits chymotrypsin-like proteasome activity [28], and inhibition of chymotrypsin-like proteasome activity is correlated with the onset of apoptosis in HeLa cells [29]. In addition, it has been demonstrated that ritonavir, present in these tablets (Aluvia and Ritoataz), displays anti-cancer properties in various other cancer types, including breast and lung cancer [30,31]. Therefore, based on our findings, the limited effectiveness of Aluvia and Ritoataz tablets in enhancing apoptosis in HeLa cells could be linked to the excipients included in their formulation. This is because excipients can interact chemically and physically with the active compounds, which may adversely affect the overall potency of the drug [32,33].

### 3.3. Pure HIV Protease Inhibitors and HIV Protease Inhibitor Tablets Induced Cell Cycle Arrest in HeLa Cervical Cancer Cells

**Figure 9:**
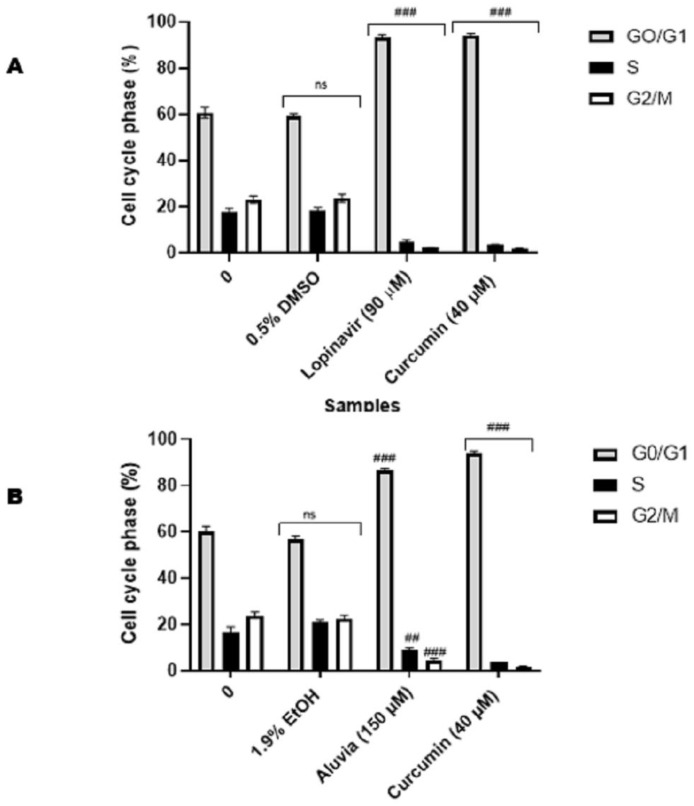

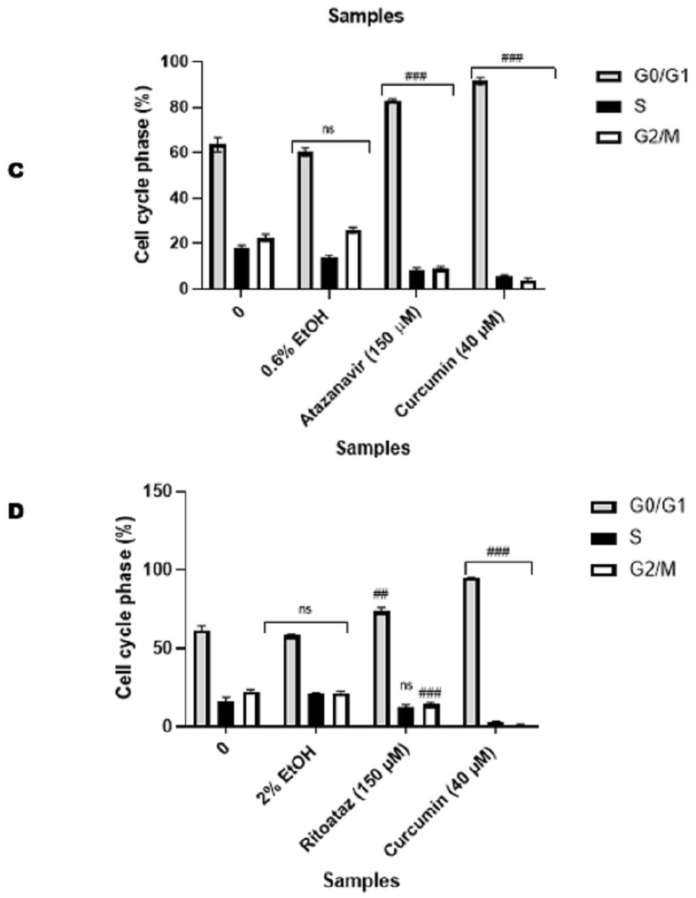
The effect of lopinavir (**A**), Aluvia (**B**), atazanavir (**C**) and Ritoataz (**D**) on cell cycle progression. The solvent controls had no significant effect (ns) on the progression of the cell cycle in HeLa cells when compared to the untreated control. All the HIV protease inhibitors significantly (*p* ≤ 0.01 ^##^/*p* ≤ 0.001 ^###^) halted the cell cycle progression at the G0/G1 phase.

Cell-cycle analysis was conducted to further understand the anticancer activities of the pure HIV-PI and tablets against the HeLa cervical cancer cells. This assay measures the quantities of cell populations in various phases of the cell cycle and at different checkpoints. The findings derived from the analysis of the cell cycle showed that treatment with 90 µM of lopinavir (Figure 9A), 150 µM of Aluvia tablets (Figure 9B), 150 µM of atazanavir (Figure 9C), and 150 µM of Ritoataz tablets (Figure 9D) resulted in the induction of G0/G1 cell cycle arrest in HeLa cells. This trend was also observed in HeLa cells treated with the positive control, 40 μM curcumin.

## 4. Discussion

There is substantial evidence suggesting that HIV-PIs not only possess antiretroviral properties but also exhibit pleiotropic pharmacological actions, which include anticancer effects [34,35]. The possible use of HIV-PIs as a novel treatment approach for cancer was first discovered through their efficacy in managing HIV-associated Kaposi’s sarcoma (KS) [34]. Although the initial assumption was that this success was due to immune system restoration and improved management of oncogenic viral infections, several studies have indicated alternative mechanisms for the antineoplastic effects of HIV-PIs in treating various tumors, such as lymphoma [36], thyroid cancer [37], multiple myeloma [38], and prostate cancer [39], thus indicating that HIV protease inhibitors may be repurposed for the development of new anticancer therapeutics. Lopinavir and its tablet formulation have exhibited efficacy against HPV 18 cervical cancer cells. However, the susceptibility of cervical cancer cells associated with HPV 18 to atazanavir, both in its standalone form and as Ritoataz tablets, remains ambiguous.

In this study, an examination was conducted on the anti-cancer properties of the HIV protease inhibitor atazanavir, as well as its tablet form (Ritoataz), in comparison to lopinavir, both in its individual form and as a tablet (Aluvia). Due to the adverse effects of chemotherapeutic agents on the kidneys, which often hinder the effectiveness of cancer treatment [40], we have chosen to employ human embryonic kidney cells (HEK-293) as a control cell line in cytotoxicity assays. This decision is based on the fact that kidneys play a crucial role in eliminating numerous antineoplastic drugs and their byproducts. Furthermore, HEK-293 cells are consistently utilized as a control cell line in in vitro studies evaluating the cytotoxic effects of drugs on breast cancer [41], lung cancer [42], and cervical cancer [43].

Over the past few years, the potency measured in in vitro assays has taken a central role in guiding the screening and selection of drug leads. Although nanomolar potency is considered advantageous, it fails to provide meaningful predictive insights in complex diseases such as cancer. The US National Cancer Institute indicates that a natural compound is considered to possess significant anticancer properties if it exhibits an IC_50_ value of approximately 10 µM or lower or 4 µg/mL [44,45,46]. Our findings indicate that atazanavir exhibits a low level of potency, necessitating a concentration of 150 micromolar to inhibit the viability of HeLa cells, which is consistent with its tablet form, Ritoataz, that similarly presents an IC_50_ of 150 micromolar. Interestingly, these drugs exhibited cytotoxic properties exclusively on HeLa cells while having no significant effect on the non-cancerous HEK-293 cells.

The role of HIV protease inhibitors in triggering apoptosis within cancer cells has been documented. Specifically, nelfinavir has been found to promote apoptosis and induce G0/G1 cell cycle arrest in liposarcoma through the upregulation of sterol regulatory element binding protein-1 [47]. In addition, saquinavir has demonstrated the ability to inhibit proteasome function, resulting in apoptosis and enhanced radiosensitivity in prostate carcinoma, a cancer that is not linked to HIV [48]. Given the role of HIV protease inhibitors in promoting apoptosis, we deemed it appropriate to investigate whether treatment with atazanavir, along with its corresponding tablets, would induce apoptosis in HPV-18 HeLa cells. Interestingly, atazanavir alone induced more apoptosis relative to Ritoataz tablets despite both exhibiting identical IC_50_ values of 150 µM. Therefore, it will be necessary to develop atazanavir derivatives, which might be highly potent and show better activity than the standard atazanavir and Ritoataz tablets. Furthermore, studies have already shown the potential of protease inhibitors as potential chemosensitizers for combination anticancer therapies [49,50]. For example, the antiviral agent cidofovir, which acts as an inhibitor of herpesvirus DNA polymerase, has been demonstrated to reduce the transcriptional expression of E6 and E7 [51]. Additionally, it was observed in the phase I clinical trial that the combination of cidofovir with cisplatin and radiotherapy led to the regression of cervical cancer tumors [50]. Therefore, our data also supports the use of atazanavir in both preclinical and clinical trials to be tested in combination with chemotherapeutic drugs to determine whether the combination of cancer chemotherapy and HAART can improve response rates compared to antineoplastic therapy alone, especially in HPV 18-related malignancies.

When comparing apoptosis induced in HeLa cells treated with atazanavir to those treated with lopinavir, lopinavir exhibited the highest induction of apoptosis. Conversely, the corresponding tablets exhibited less apoptosis-inducing efficacy. Since the induction of apoptosis in HPV 18 HeLa cells was not predominantly observed after treatment with Aluvia and Ritoataz tablets, it is possible that the excipients included during the formulation of these tablets may affect the apoptotic effects of the individual PIs within these tablets. This is possible because a study by Kurmi et al. [52] showed that the interaction between abacavir sulfate, an antiviral medication, and lactose monohydrate, an excipient, resulted in the generation of two new products. This outcome suggested a degree of incompatibility that could negatively impact both the bioavailability and stability of the abacavir sulfate drug. In addition, since certain factors that trigger cell cycle arrest also play a role in initiating apoptosis, our next objective was to investigate the effect of HIV protease inhibitors and their respective tablets on the progression of the cell cycle in HeLa cells. Interestingly, G0/G1 cell cycle arrest was observed in all the drugs, aligning with previous research that demonstrated comparable outcomes across various cancer types [35,53,54]. An arrest in the G1 phase of the cell cycle provides cells with a chance to either undergo repair mechanisms or enter the apoptotic pathway. Based on our findings, the G1-phase arrest is associated with apoptosis, thereby indicating that HeLa cells are indeed sensitive to HIV protease inhibitors.

## 5. Conclusions

Ever-present hurdles for the discovery of new drugs for HPV-related cancer therapy have necessitated the repurposing of already manufactured drugs for novel therapeutic purposes. The findings of this study demonstrated that HIV-PIs affect cell viability, apoptosis, and cell cycle progression, suggesting their potential in treating HPV-related cancers. Nevertheless, similar to the findings associated with lopinavir in in vitro settings, both atazanavir and Ritonavir tablets exhibit low potency. Our findings underscore the necessity for the development of atazanavir derivatives that may possess enhanced potency and demonstrate higher activity compared to the standard atazanavir. Furthermore, our data suggest that atazanavir could be effectively combined with chemotherapeutic agents to evaluate whether such combinations will yield improved response rates relative to antineoplastic therapy alone.

## Figures and Tables

**Table 1:** Statistical analysis of the total apoptosis induced by HIV-PIs in HeLa cells.

	Mean (%) ± SEM	
	Live	Total Apoptosis
150 µM atazanavir	54.842 ± 2.668	36.717 ± 1.729 ***
150 µM Ritoataz	66.837 ± 5.329	28.432 ± 2.374 ***
90 µM lopinavir	51.217 ± 2.616	41.583 ± 1.001 ***
150 µM Aluvia	70.258 ± 1.537	26.795 ± 0.6805 ***

***: *p* ≤ 0.001.

## Data Availability

Data are contained within the article.
